# Complete pathological response in patients with *HER2* positive breast cancer treated with neoadjuvant therapy in Colombia

**DOI:** 10.7705/biomedica.6665

**Published:** 2023-09-30

**Authors:** Mauricio Rodríguez, Diego M. González, Farah El-Sharkawy, Mileny Castaño, Jorge Madrid

**Affiliations:** 1 Departamento de Cirugía Oncológica, Universidad de Antioquia, Medellín, Colombia Universidad de Antioquia Universidad de Antioquia Medellín Colombia; 2 Instituto de Cancerología Las Américas Auna, Medellín, Colombia Instituto de Cancerología Las Américas Auna Medellín Colombia; 3 Department of Pathology, University of Pennsylvania Perelman School of Medicine, Philadelphia, PA, United States of America University of Pennsylvania University of Pennsylvania Perelman School of Medicine Philadelphia PA USA

**Keywords:** Breast neoplasms, neoadjuvant therapy, Colombia, Neoplasias de la mama, terapia neoadyuvante, Colombia

## Abstract

**Introduction.:**

Breast cancer is the most common type of cancer and the leading cause of death by cancer in women in Colombia. Approximately 15 to 20% of breast cancers overexpress *HER2*.

**Objective.:**

To analyze the relationship between multiple clinical and histological variables and pathological complete response in patients with *HER2*-positive breast cancer undergoing neoadjuvant therapy in a specialized cancer center in Colombia.

**Materials and methods.:**

We performed a retrospective analysis of non-metastatic *HER2*- positive breast cancer patients who received neoadjuvant therapy between 2007 and 2020 at the *Instituto de Cancerología Las Americas Auna* (Medellín, Colombia). Assessed parameters were tumor grade, proliferation index, estrogen receptor, progesterone receptor, *HER2* status, type of neoadjuvant therapy, pathologic complete response rates, and overall survival.

**Results.:**

Variables associated with low pathologic complete response rates were tumor grades 1-2 (OR = 0.55; 95% CI = 0.37-0.81; p = 0.03), estrogen receptor positivity (OR = 0.65; 95%; CI = 0.43-0.97; p=0.04), and progesterone receptor positivity (OR = 0.44; 95% CI = 0.29-0.65; p = 0.0001). *HER2* strong positivity (score 3+) was associated with high pathological complete response rates (OR = 3.3; 95% CI = 1.3-8.35; p=0.013). Five-year overall survival was 91.5% (95% CI = 82.6-95.9) in patients with pathological complete response and 73.6% (95% CI = 66.4-79.6) in patients who did not achieve pathological complete response (p = 0.001). Additionally, the pathological complete response rate was three times higher in patients receiving combined neoadjuvant chemotherapy with anti- *HER2* therapy than in those with chemotherapy alone (48% versus 16%).

**Conclusion.:**

In patients with *HER2*-positive breast cancer, tumor grade 3, estrogen receptor negativity, progesterone receptor negativity, strong *HER2* positivity (score 3+), and the use of the neoadjuvant trastuzumab are associated with higher pathological complete response rates.

## Resumen

**Introducción.:**

El adenocarcinoma de seno es el tipo de cáncer más frecuente y con mayor tasa de mortalidad asociada en mujeres en Colombia. Aproximadamente entre el 15 al 20 % de estos cánceres sobreexpresan el gen *HER2*.

**Objetivo.:**

Analizar las asociaciones existentes entre múltiples variables clínicas e histológicas con respecto a la respuesta patológica completa en pacientes con cáncer de mama *HER2* positivo que fueron tratadas con quimioterapia neoadyuvante en un centro especializado en el tratamiento del cáncer en Colombia.

**Materiales y métodos.:**

Se realizó un análisis retrospectivo de las pacientes con cáncer de mama *HER2* positivo, no metastásicas, que recibieron quimioterapia neoadyuvante entre el 2007 y el 2020 en el Instituto de Cancerología Las Américas Auna (Medellín, Colombia). Se evaluaron los parámetros de grado tumoral, índice de proliferación, estatus de receptores de estrógeno y de progesterona, tipo de quimioterapia neoadyuvante recibida, tasas de respuesta patológica completa y supervivencia global.

**Resultados.:**

Las variables asociadas con tasas de respuesta patológica completa más bajas fueron grados tumorales 1-2 (OR = 0,55; IC 95% = 0,37-0,81; p= 0,03), positividad de receptores de estrógeno (OR = 0,65; IC 95 % = 0,43-0,97; p = 0,04) y positividad de receptores de progesterona (OR = 0,44; IC 95 % = 0,29-0,65; p = 0,0001). La positividad fuerte para *HER2* (puntaje 3+) se asoció a tasas de respuesta patológica completa más altas (OR = 3.3; IC 95 % = 1,3-8,35; p = 0,013). La supervivencia global a cinco años fue del 91,5 % (IC 95 % = 82,6-95,9) en pacientes con respuesta patológica completa y del 73,6 % (IC 95 % = 66.4-79.6) en pacientes sin respuesta patológica completa (p = 0.001). La tasa de respuesta patológica completa fue tres veces mayor en los pacientes que recibieron quimioterapia neoadyuvante con terapia anti-*HER2* comparado con aquellos que recibieron quimioterapia sola sin agentes anti-*HER2* (48 % versus 16 %).

**Conclusión.:**

En pacientes con cáncer de mama con sobreexpresión de *HER2*, grado tumoral tres, receptores de estrógeno y progesterona negativos, positividad fuerte para *HER2* (puntaje 3+) y uso de quimioterapia neoadyuvante con trastuzumab se asociaron con mayores tasas de respuesta patológica completa.

Breast cancer is the most common type of cancer and the leading cause of death by cancer in women in Colombia. Approximately, 13,000 new cases and 3,700 deaths are reported annually nationwide ^1^^-^^3^. Data from the Antioquia population cancer registry shows that breast cancer represents 17 % of the total cancer cases in this Colombian region. It is the type of cancer with the highest prevalence and accounts for 13.5 % of mortality by cancer, only superseded by lung cancer ^4^^-^^7^.

North American data shows significantly improved overall survival and disease-free survival of breast cancer in the last decade, likely due to better treatment strategies guided by immunohistochemical subclassification ^8^^,^^9^. Using immunohistochemical for estrogen receptor, progesterone receptor, human epidermic growth factor receptor 2 (*HER2*), and Ki-67 (proliferation rate), breast cancer can be divided into the following subtypes ^10^^,^^11^:

- ***Luminal A:*** estrogen receptor positive, progesterone receptor positive, *HER2* negative, and low Ki-67.
- ***Luminal B1:*** estrogen receptor positive, *HER2* negative, and progesterone receptor negative or high Ki-67.
- ***Luminal B2:*** estrogen receptor positive and *HER2* positive, regardless of progesterone receptor or Ki-67.
- ***HER2 enriched:*** *HER2* positive and estrogen receptor negative.
- ***Triple-negative:*** estrogen receptor negative, progesterone receptor negative, and *HER2* negative.

Notably, approximately 15 to 20% of breast cancers overexpress *HER2*.

The standard treatment worldwide is the administration of neoadjuvant chemotherapy or targeted anti-*HER2* therapy in the following cases ^12^:

1. Patients with locally advanced disease: N+ (independent of tumor stage) or higher than T3 independent of the immunohistochemical subgroup.
2. Patients with tumor stage higher than T1c triple negative or *HER2* positive cancer ^13^.
3. Patients with a high tumor-to-breast volume ratio, with possible future breast-conserving surgery.

Neoadjuvant therapy in *HER2*-positive tumors has the benefit of increased rates of breast-conserving surgery without compromising ^14^ or even improving long-term oncological outcomes such as overall survival and recurrence ^15^^,^^16^. Adding anti-*HER2* antibody therapies, such as trastuzumab, to neoadjuvant chemotherapy not only doubles pathological complete response rates but is also associated with improved disease-free survival ^17^^,^^18^.

The pattern of response to neoadjuvant therapy is also important. The CTNeoBC study showed that pathological complete response in the breast and axillae was related with increased disease-free survival and overall survival compared to pathological complete response in the breast only. This association was strongest in triple-negative and *HER2*-enriched groups, decreasing the probability of death by 84% and 71%, respectively ^19^.

In Colombia, there is not sufficient data regarding the pathological complete response of *HER2*-positive breast cancer after neoadjuvant therapy.

This study aimed to analyze the relationship between multiple clinical and histologic variables and pathological complete response status in patients with *HER2*-positive breast cancer undergoing neoadjuvant therapy in a specialized cancer center in Colombia.

## Materials and methods

We performed a retrospective analysis of 467 non-metastatic *HER2*- positive breast cancer patients who received neoadjuvant therapy between 2007 and 2020 at the *Instituto de Cancerología Las Américas Auna* (Medellín, Colombia).

Data was obtained from the institutional cancer registry RIC REDCAP. The exclusion criteria were age under 14 or above 75 years, the presence of a second primary malignancy in the last five years (except for non-melanoma skin cancer), less than six months of life expectancy after initiation of neoadjuvant therapy, and patients missing 25% or more data.

Pathological complete response was defined as the absence of invasive carcinoma in the breast and lymph nodes after neoadjuvant therapy. Overall survival was defined as the percentage of patients who are still alive after a period of five years, and disease-free survival was defined as the length of time after treatment that the patient survives without any signs or symptoms of breast cancer.

Quantitative variables were presented as mean and standard deviation (SD) or interquartile range (IQR). Categorical variables were presented as absolute values or percentages. The Kolmogorov-Smirnov test was used to examine normality. Bivariate analysis of continuous and categorical variables was performed using t test, Mann Whitney U, and chi square test, as appropriate, and associations were measured with odds ratios (OR). A p-value less than or equal to 0.05 was considered statistically significant. Differences in overall survival were assessed using the Kaplan-Meier method and compared with the log-rank test. All analyses were performed with Stata, version 12 software (StataCorp, College Station, TX, USA).

This study received approval from the institutional review board of *Instituto de Cancerología Las Américas Auna* and was conducted following the 1964 Helsinki Declaration and its later amendments.

## Results

**Figure 1:**
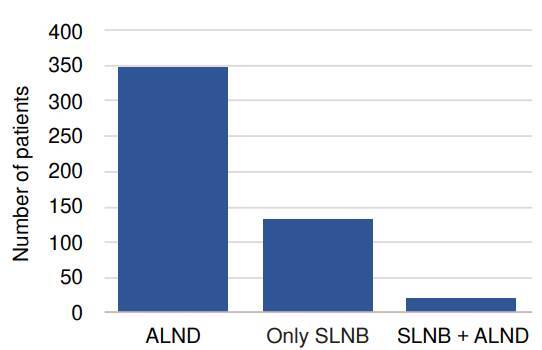
Number of patients by type of axillary surgery

ALND: *Axillary lymph node dissection*; SLNB: *Sentinel lymph node biopsy*

A cancer registry review yielded 467 patients that met the study criteria. Demographic, clinical, and histologic characteristics of the study population are summarized in table 1. The mean age was 50 years, the mean body mass index was 26.9, and the mean size of the lesion before neoadjuvant therapy was 48 mm. A total of 43.5% of patients had a family history of breast cancer. The most common stage at presentation was stage III, with 54.2% of patients, followed by stage II (34.9%) and stage I (7.5%). Axillary lymph node dissection was performed in 69% of patients, sentinel lymph node biopsy in 31%, mastectomy in 66%, and breast-conserving surgery in 34% (figure 1).

**Table 1:** Demographic, clinical, and histological characteristics of the studied population

**Variable**		**Number (N=467)**
Age (years), mean (IQR)		50 (22-75)
Weight (kg), mean (IQR)		65.7 (35-114)
Body Mass Index, n (%)		
	Mean	26.9
	<20	11 (24)
	21-25	167 (35.8)
	26-30	173 (37)
	>30	113 (24.2)
T status		
	T1	45 (9.6)
	T2	173 (37)
	T3	82 (17.6)
	T4	166 (35.5)
N status		
	N0	138 (29.6)
	N1	241 (51.6)
	N2	73 (15.6)
	N3	9 (1.9)
ER (n=421)		
	Positive	270 (64.1)
	Negative	151 (35.9)
PR (n=418)		
	Positive	232 (55.5)
	Negative	186 (44.5)
HER2 (n=378)		
	3+	350 (92.6)
	2+ (FISH +)	28 (7.4)
Ki67 (n=342)		
	<20	38 (11.1)
	≥20	304 (88.8)
Histological grade (n=459)		
	1	44 (9.6)
	2	163 (35.5)
	3	252 (54.9)
Lymphovascular invasion (n=467)		
	Yes	72 (15.4)
	No	395 (84.6)
pCR, n (%) (n=467)		
	Yes	184 (39)
	No	283 (61)

QR: *Interquartile range*; T: *Tumour size*; N: *Lymph node*; ER: *Estrogen receptor*; PR: *Progesterone receptor*; pCR: *Pathological complete response*

The associations of different clinical and histological variables with pathological complete response status are shown in table 2. Variables associated with low pathological complete response rates were tumor grades 1-2 (OR=0.55; 95% CI=0.37-0.81; p=0.03), estrogen receptor positivity (OR=0.65; 95% CI=0.43-0.97; p=0.04), and progesterone receptor positivity (OR=0.44; 95% CI=0.29-0.65; p=0.0001). In contrast, strong *HER2* positivity (score 3+) was associated with high pathological complete response rates (OR=3.3; 95% CI=1.3-8.35; p=0.013).

**Table 2:** Associations of clinical and histologic variables with pCR status

Variable		pCR (n)	Non pCR (n)	Total (n)	OR (95% CI)	p value
Age (years)						
	<40	26	51	77	0.74 (0.44-1.25)	0.32
	>40	158	232	390		
Histologic grade						
	1 and 2	65	142	207	0.55 (0.37-0.81)	0.0003
	3	114	138	252		
Ki 67						
	<20	14	24	38	0.62 (0.31-1.25)	0.24
	≥ 20	147	157	304		
ER						
	Positive	104	166	270	0.65 (0.43-0.97)	0.47
	Negative	74	77	151		
PR						
	Positive	79	153	232	0.44 (0.29-0.65)	0.0001
	Negative	100	86	186		
*HER2*						
	3+	166	184	350	3.30 (1.30-8.35)	0.013
	2+ (FISH +)	6	22	28		
Surgery window time (days)						
	<15	7	10	17	1.00	0.07
	16-45	70	91	161	0.91	
	46-90	84	127	211	1.05	
	91-180	21	31	52	1.03	
	>180	0	12	12		

ER: *Estrogen receptor*; PR: *Progesterone receptor*; pCR: *Pathological complete response*; OR: *Odds ratio*

**Figure 2:**
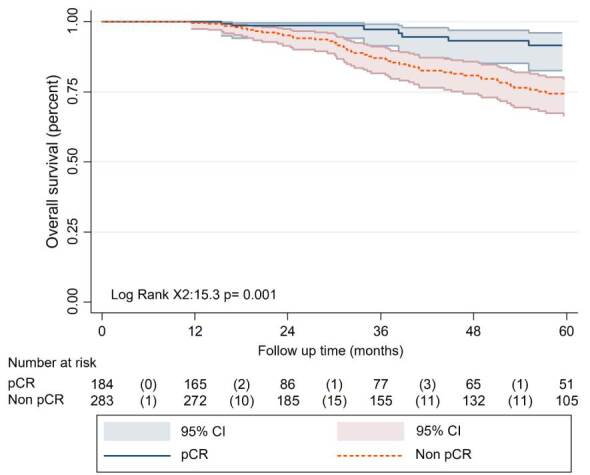
Survival analysis based on pathologic complete response status

The average five-year overall survival was 79.1% (95% CI=73.5-83.6), with a median follow-up of 35 months. In the group that achieved pathological complete response, 168 patients (91.5%; 95% CI=82.6-95.9) were alive after five years, compared with 208 patients (73.6%; 95% CI=66.4-79.6) in the non-pathological complete response group (figure 2). This difference was statistically significant (p=0.001).

pCR: *Pathological complete response*

**Figure 3:**
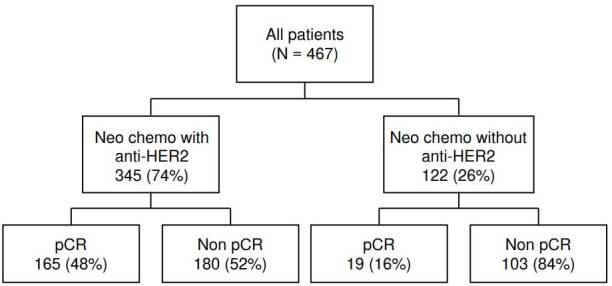
Number of patients by type of neoadjuvant therapy and rates

Ninety patients were initially classified as *HER2*-negative based on biopsy results and were later classified as *HER2*-positive based on examination of the resection specimen. Combined neoadjuvant chemotherapy and anti- *HER2* therapy (trastuzumab) were administered to 345 patients (74% of the total), while the remaining 122 (26%) patients received neoadjuvant chemotherapy alone (figure 3). The latter included those 90 patients whose initial biopsy was *HER2*-negative, and the patients who received their first and second chemotherapy cycles at other healthcare institutions. None of the patients received neoadjuvant radiotherapy. The pathological complete response rate was three times higher in patients receiving combined neoadjuvant chemotherapy with anti-*HER2* therapy than in those with chemotherapy alone (48% versus 16%).

pCR: *Pathological complete response; HER2: Human epidermal growth factor receptor 2*

### Ethical approval

This study was performed in line with the principles of the Declaration of Helsinki. Approval was granted by the Ethics Committee of the *Instituto de Cancerología Las Américas Auna*.

## Discussion

The most used pathological complete response definition is the absence of residual invasive cancer in the breast and lymph nodes after neoadjuvant therapy ^20^^,^^21^. In our study, tumor grade 3, estrogen receptor negativity, progesterone receptor negativity, *HER2* strong positivity (score 3+), and the use of neoadjuvant trastuzumab were associated with higher pathological complete response rates. Unsurprisingly, a significantly increased overall survival was observed in patients with pathological complete response. The findings of this study are of particular importance for the Colombian population since oncological guidelines and protocols employed in this country have been largely based on studies conducted elsewhere. However, our findings agree with the international literature and confirm previously reported results.

It has been well documented that anti-*HER2* monoclonal antibodies (e.g., trastuzumab) improve overall survival (primary goal) and pathological complete response rates (surrogate result) in patients with luminal B2 or *HER2*-enriched breast cancers ^22^. Based on our data, the number needed to treat with trastuzumab, to achieve pathological complete response, was three (16% versus 48% pathological complete response rate). This agrees with results from the phase 3 trial NOAH ^18^, which reported a 38% pathological complete response rate in patients receiving combined neoadjuvant chemotherapy with trastuzumab, compared with a 19% pathological complete response rate in patients with chemotherapy alone (number needed to treat of five, p=0.001). Results from this trial also showed increased five-year overall survival and disease-free survival in the combined chemotherapy with trastuzumab group (overall survival 74% versus 63%; hazard ratio=0.66; p=0.055). In a pooled analysis by Petrelli *et al*. ^23^, the addition of trastuzumab to neoadjuvant chemotherapy decreased the relapse rate from 20 to 12% and increased the pathological complete response rate from 20 to 43% (risk ratio=2.07; 95% CI=1.41-3.30; p=0.0002). Similarly, other studies have demonstrated that adding trastuzumab to neoadjuvant chemotherapy in *HER2*-positive breast cancer patients was associated with a threefold increase in pathological complete response rates ^24^^,^^25^.

In a pooled analysis of almost 6,500 patients with breast cancer receiving neoadjuvant therapy ^13^, pathological complete response was associated with increased disease-free survival (hazard ratio=0.446), and this correlation was greater in patients with triple-negative or *HER2*-positive breast cancers. The CTNeoBC study, including almost 12,000 patients, showed that pathological complete response in the breast and axillae was associated with increased disease-free survival and overall survival compared to pathological complete response only in the breast ^19^. The NeoSphere trial reported a five-year disease-free survival of 75% in non-pathological complete response patients compared with 85% in pathological complete response patients receiving neoadjuvant dual *HER2* blockade ^22^. These results were confirmed by the phase 2 TRYPHAENA trial, which demonstrated that neoadjuvant chemotherapy combined with dual *HER2* blockade (trastuzumab + pertuzumab) was associated with increased pathological complete response rates, from 55 to 64%, compared to neoadjuvant chemotherapy plus trastuzumab only ^26^^,^^27^. This effect was greater in patients with estrogen receptor-negative disease ^27^^,^^28^.

Based on the data above, recommendations from the St. Gallen and ESMO guidelines ^11^^,^^29^ include neoadjuvant chemotherapy plus *HER2* blockade in patients with *HER2*-positive breast cancer. Trastuzumab use should be continued for one year after surgery (adjuvant therapy). Following the KATHERINE trial ^14^, emtansine (T-DM1) use should be considered in patients who fail to achieve a pathological complete response. This phase 3 trial compared 14 cycles of either T-DM1 or trastuzumab as adjuvant therapy in patients with residual invasive disease following trastuzumabbased neoadjuvant therapy. They found a reduced risk of invasive and distant recurrence and an increased invasive-disease-free survival at three years, from 77.0 to 88.3% (hazard ratio = 0.50; p<0.001). The St. Gallen and ESMO guidelines also recommend using dual *HER2* blockade in the neoadjuvant setting and for one year as adjuvant therapy.

In our study, an unexpectedly large number of patients (approximately 20%) were deemed *HER2*-negative at initial diagnosis based on biopsy results. Previous studies suggest that around 3% of patients initially classified as *HER2*-negative are later diagnosed as *HER2*-positive following surgical resection ^30^. Our findings may be explained by possible technical issues in the staining procedure or the interpretation of *HER2* and suggest that tissue handling and biopsy review by specialized pathology practices must be warranted before making treatment decisions.

This study has several limitations. One of the major disadvantages is the observational and retrospective nature of the study because, despite the size of the sample (one of the most extensive series documented in this population), the results cannot be generalized to all of Colombia due to the biases it could have. In addition, pathological complete response was used as the primary outcome, but its definition is not universal. Furthermore, pathological complete response has emerged as an early endpoint for clinical trials investigating novel approaches to decrease the time needed to evaluate these therapies, and it is a well-known surrogate result for long-term outcomes. In a future analysis, we plan to report the association of specific chemotherapeutic agents with long-term outcomes, such as overall survival, disease-free survival, and pathological complete response.

In summary, our study shows that patients with *HER2*-positive breast cancer, tumor grade 3, estrogen receptor negativity, progesterone negativity, strong *HER2* positivity (score 3+), and the use of neoadjuvant trastuzumab have higher pathological complete response rates. Pathological complete response is associated with increased overall survival.
